# Case report: An EEG captured case of migralepsy/migraine aura-triggered seizures

**DOI:** 10.3389/fneur.2022.953224

**Published:** 2022-08-11

**Authors:** Anam Hareem, Mahsa Pahlavanzadeh, Nicholas E. Calvo, Sanaz Monjazeb, Chinekwu Anyanwu

**Affiliations:** ^1^Atrium Health Wake Forest Baptist Medical Center, Winston-Salem, NC, United States; ^2^Department of Neurology, Carilion Clinic, Roanoke, VA, United States; ^3^Department of Neurology, The University of Utah, Salt Lake City, UT, United States; ^4^Department of Neurology, University of Southern California, Los Angeles, CA, United States; ^5^Department of Neurology, Virginia Tech Carilion, Roanoke, VA, United States

**Keywords:** migralepsy, visual aura, ictal headache, homonymous hemianopsia, visual hallucinations

## Abstract

**Introduction:**

Migraine and epilepsy are common chronic neurological disorders presenting with paroxysmal attacks of transient cerebral dysfunction, followed by subsequent return to baseline between episodes. The term “migralepsy” has been proposed to define migraine-triggered epileptic seizures classified by the ICHD-III as a complication of migraine with an aura.

**Case:**

A 55-year-old man with a 30-year history of migraine without aura presented with a new onset left parietal pain accompanied by visual disturbances occurring up to 20 times per day. His visual distortions included kaleidoscopic vision, flashes of shadows, and a right superior quadrantanopia lasting 20 min. He described discrete 2-min episodes of scintillating scotomas in his right visual field. Ictal EEG demonstrated a left occipital onset focal aware seizure with his clinical symptoms. The patient was started on valproic Acid and has remained asymptomatic.

**Discussion:**

The diagnostic criteria as set out by the ICHD-III for migralepsy and other syndromes with migrainous and ictal features remain confusing for practitioners as there is much overlap in clinical manifestations of these entities. EEG should be obtained when ictal features are noted among patients presenting with headache.

## Introduction

Epilepsy and migraine are the most encountered chronic neurologic disorders associated with paroxysmal attacks of transient cerebral dysfunction, followed by subsequent return to baseline between episodes. Migraine and epilepsy both share a heritable component, demonstrate high rates of comorbidity, have similar underlying pathophysiological mechanisms, and overlap in symptomatology. In these conditions, we see excessive neuronal excitability characterized by visual and sensory disturbances, autonomic symptoms, and, at times, alterations in the content and level of consciousness (1–3). Although studies vary, individuals with either migraine or epilepsy are more than twice as likely to have the other disorder. The prevalence of migraine in the epileptic population ranges between 8.4 and 23% (4) while the prevalence of epilepsy in people with migraine varies from 1 to 17% (5). The lack of published data highlights the inadequacy of the current definitions of ICHD-III about temporal and/or clinical association and overlap between migraine and epilepsy. Herein we present a case in which symptoms of the usual migraine aura developed into a focal aware seizure of occipital origin.

## Case

A 55-year-old man with a known history of migraine headaches without aura, hypertension, hyperlipidemia, obesity, and newly diagnosed type 2 diabetes mellitus II was referred to the Emergency Department (ED) by his ophthalmologist. The patient had a 5-day history of visual disturbances and a severe left parietal headache. He described the quality of his headache as lancinating, continuous, and pulsatile. He described his visual distortions as having a “wavy quality” with associated kaleidoscopic patterns, flashes of shadows, and scintillating scotomas (red and green circles), occurring every 15–20 min and lasting 2 min each. His visual field was also obscured in the right hemifield, more so in his right superior quadrant.

The onset of his symptoms was associated with nausea, vomiting, and intermittent sensitivity to light. He reportedly received chiropractic manipulation hours earlier in the day prior to the onset of his symptoms. He denied sensitivity to sound or smell, or other neurologic deficits including slurred speech, double vision, focal weakness, numbness, lacrimation, painful eye movements, or nuchal rigidity. The frequency of his typical migraine headaches occurred one to two times per year.

His neurologic exam showed a right superior homonymous quadrantanopia with 20/25 visual acuity in both eyes, full ocular motility, and unremarkable funduscopy. The initial work up included computed tomographic angiography (CTA) of the head and neck, contrast-enhanced magnetic resonance imaging (MRI) of the brain and orbit with and without contrast, and blood work including erythrocyte sedimentation rate (ESR), C-reactive protein (CRP), and complete blood count (CBC), which were all normal. The patient was discharged from the ED with resolution of his headache and visual symptoms. He was advised to obtain an contrast enhanced MRI Orbit with and without contrast and follow up at the in ophthalmology and neurology clinics in 1 week.

**Figure 1:**
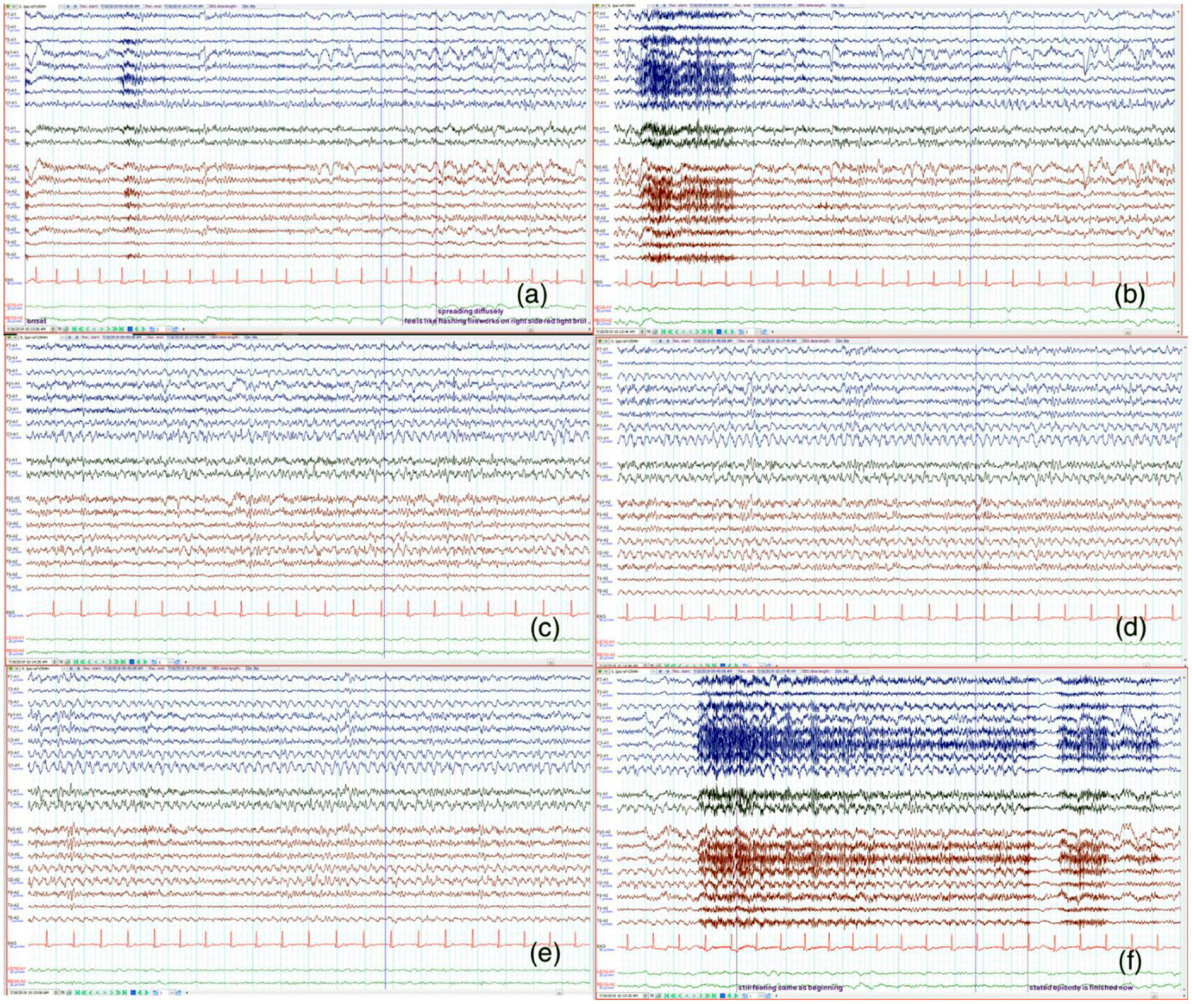
The signals from the left hemisphere is shown in blue and right hemisphere in red. Twenty sec epochs of electroencephalography (EEG) (average montage) demonstrate that the onset is from the left occipital region **(a)** with evolution and progression **(b–f)** to involve the right side. The patient's seizure correlated with the visual sensation of “flashing fireworks and broken red lights mirrors” in the patient's right visual hemifield.

He returned to the hospital 5 days later due to more frequent episodes of visual distortion. He reported that the headache was no longer prominent compared to his initial presentation a week prior and the visual field deficit had resolved. Electroencephlogram (EEG) was suggested given the unremarkable results of the previous workup and his persistent visual symptoms. An initial short-term EEG was normal. A typical event was captured during the long-term recording. Ictal EEG demonstrated a left occipital-onset focal seizure with right hemispheric involvement as it evolved (Figure 1). This correlated with the patient reporting “flashing fireworks and broken red lights mirrors” in the right eye.

He was loaded with levetiracetam 2000 mg intravenously with a daily maintenance oral dose of levetiracetam 750 mg twice daily. On this regimen, he continued to report several episodes of the visual disturbance the following next day. He was switched to valproic acid (loading dose of 2000 mg and maintenance dose of 500 mg twice daily). A repeat routine EEG the following day was normal. Upon his 2-month follow up with neurology, he reported resolution of the headaches and visual disturbances.

## Discussion

The term migralepsy was first coined in 1960 by Lennox and Lennox to describe a condition wherein “ophthalmic migraine with perhaps nausea and vomiting was followed by symptoms characteristic of epilepsy” (6). The International Classification of Headache Disorders-III (ICHD-III) has refined the definition of migralepsy to denote an attack of migraine with aura attack that is complicated, by an epileptic seizure within an hour of migraine onset (7). Currently, it is better known as migraine-triggered seizure and is a rare phenomenon. Within the same family of conditions, other rare neurological disorders with migrainous and ictal components include including ictal epileptic headaches (IEH) and hemicrania epileptica. The ICHD-III refers to IEHs as secondary headache disorders to a primary epileptic condition and occurs in 3–5% of cases (8, 9). The headache can be ipsilateral or contralateral with epileptiform EEG discharges. The original diagnostic criteria for ictal epileptic hedaache as proposed by Parisi et al. also includes a prompt response to antiepileptic drugs (10). Hemicrania epileptica is recognized as an ipsilateral “ictal headache” that occurs “synchronously” with a partial seizure. To complicate matters further, there have been reports of ictal epileptic headaches that present as the sole manifestation of an epileptic event, thus making the differentiation of migraine with aura from epileptic seizures difficult (11–13).

Our case demonstrates yet another example of the complex relationship between migraine and epilepsy. Identifying the correct underlying etiology of the patient's symptoms was difficult because of the fluctuating nature of his complaints and deficits seen on neurologic exam. In this case, migraine-like symptoms appeared first followed by his visual disturbances. However, as his symptoms persisted, the visual disturbances became more prominent and were suspicious for occipital onset seizures. By ICHDIII criteria, this patient would qualify for diagnosis of migraine-triggered seizure/migralepsy because the initial migraine headaches triggered his focal aware left occipital lobe seizures. However, over the course of the patient's disease, the visual disturbances associated with his seizures became the prominent feature and the headaches had resolved, thus obscuring the picture further.

Among 50 potential migralepsy cases identified in the literature, only two were found to meet ICHD criteria. Most diagnoses remaining uncertain and often questionable due to insufficient data collected at the time of diagnosis, including diagnostic EEG studies during the event (14). In the questionable cases, the description of the episode combined with EEG and brain imaging were highly suggestive of epileptic seizures particularly in the occipital lobe (15). Our patient case was fortunate because an epileptic event was documented when EEG was recorded. Notably, apart from the seizure captured on EEG, there were no focal abnormalities or interictal epileptiform discharges seen within the recording or on a follow-up EEG (obtained 2 days after initiating proper treatment) to suggest a focal predisposition to seizure in that region. To complicate differentiating the diagnosis patients with epileptic visual symptoms may show similar EEG abnormalities to those as seen in acute migraine attacks, with posterior predominant theta-delta wave activity (15). In most cases of ictal epileptic headache ictal there is a lack of a specific headache pattern with evidence of non-specific ictal EEG patterns often without corical-topographic correlations (16).

When evaluating attacks suggestive of migralepsy one must consider the characteristics of visual symptoms between migraine visual auras and occipital lobe seizures. A retrospective cohort analysis found that the visual aura was of shorter duration in epilepsy compared to migraine, with a median duration of 56 s vs. 20 min, respectively. Other factors that differentiate the quality of the visual aura in epilepsy vs. migraine included a restriction of the visual phenomenon to a visual hemifield in epilepsy compared to migraine; and centrifugal or centripetal spread of visual symptoms (phosphenes, scotomas, etc.) occurred in migraine, but not in epilepsy. The patient described visual auras which were a combination of those typical for both migraine and epilepsy making a distinction between them a diagnostic challenge. He described a global visual kaleidoscopic pattern expected in migraine. However, the distinct flashes of shadows and scintillating scotomas described as red and green circles restricted to his right visual field of both eyes, lasting only 2 min, are more typical for epilepsy. Though there are minor differences in the quality and timing of the visual aura, an EEG was required to make a more definitive distinction. Even obtaining long-term EEG monitoring for precise diagnosis may be necessary in resolving diagnostic uncertainty (17–19). Visual auras in migraine and epilepsy are related to transient occipital lobe dysfunction which has been described in the literature as cortical spreading depression and paroxysmal depolarizing shift, respectively (19).

The therapeutic benefit of certain antiepileptic drugs (AEDs) in migraine prevention can be reasonably inferred from this shared pathogenesis with epilepsy (20, 21). The goal of management is to treat migraine and epilepsy. This can be achieved with medications including valproic acid, topiramate, or zonisamide (second line) for patients who are intolerant to topiramate (22). Our patient showed incomplete response to levetiracetam and was thus shifted to valproic acid 500 mg twice daily with complete resolution of visual symptoms and normal repeat EEG.

## Data availability statement

The original contributions presented in the study are included in the article/supplementary material, further inquiries can be directed to the corresponding author/s.

## Ethics statement

Written informed consent was obtained from the individual(s) for the publication of any potentially identifiable images or data included in this article.

## Author contributions

AH contributed to discussion and final proof reading. MP contributed to writing the case and introduction and final proof reading. NC wrote the case section. SM wrote the introduction and abstract. CA contributed to EEG discussion and proof reading. All authors contributed to the article and approved the submitted version.

## Conflict of interest

The authors declare that the research was conducted in the absence of any commercial or financial relationships that could be construed as a potential conflict of interest.

## Publisher's note

All claims expressed in this article are solely those of the authors and do not necessarily represent those of their affiliated organizations, or those of the publisher, the editors and the reviewers. Any product that may be evaluated in this article, or claim that may be made by its manufacturer, is not guaranteed or endorsed by the publisher.
